# Gut microbiome is affected by gut region but robust to host physiological changes in captive active-season ground squirrels

**DOI:** 10.1186/s42523-021-00117-0

**Published:** 2021-08-13

**Authors:** Kirsten Grond, Courtney C. Kurtz, Jasmine Hatton, Michelle M. Sonsalla, Khrystyne N. Duddleston

**Affiliations:** 1 Department of Biological Sciences, College of Arts and Sciences, University of Alaska Anchorage, 3211 Providence Dr., Anchorage, AK 99508 USA; 2 Department of Biology, College of Letters and Science, University of Wisconsin-Oshkosh, 800 Algoma Blvd., Oshkosh, WI 54901 USA

**Keywords:** Hibernation, *Ictidomys tridecemlineatus*, Firmicutes:Bactoidetes ratio, Gastrointestinal tract

## Abstract

**Background:**

Thirteen-lined ground squirrels (*Ictidomys tridecemlineatus*) are obligate hibernators and are only active 4–5 months annually. During this period, squirrels rapidly acquire fat for use during hibernation. We investigated how the gut microbiome changed over the active season in the mucosa and lumen of two gut sections: the cecum and ileum. We sequenced the 16S rRNA gene to assess diversity and composition of the squirrel gut microbiome and used differential abundance and network analyses to identify relationships among gut sections.

**Results:**

Microbial composition significantly differed between the cecum and ileum, and within the ileum between the mucosa and lumen. Cecum mucosa and lumen samples did not differ in alpha diversity and composition, and clustered by individual squirrel. Ileum mucosa and lumen samples differed in community composition, which can likely be attributed to the transient nature of food-associated bacteria in the lumen. We did not detect a shift in microbiome diversity and overall composition over the duration of the active season, indicating that the squirrel microbiome may be relatively robust to changes in physiology.

**Conclusions:**

Overall, we found that the 13-lined ground squirrel microbiome is shaped by microenvironment during the active season. Our results provide baseline data for new avenues of research, such as investigating potential differences in microbial function among these physiologically unique gut environments.

**Supplementary Information:**

The online version contains supplementary material available at 10.1186/s42523-021-00117-0.

## Background

Gut microbiome composition and diversity have been identified as important players in regulation of body weight and energy homeostasis through several digestive functions, such as enabling metabolism of complex polysaccharides and production of short-chain fatty acids through fermentation [1–4]. A majority of literature focuses on weight gain induced by diet or microbiome manipulations in humans and animal model systems like laboratory-bred mice and rats [5, 6]. Although informative, the artificial nature of such studies is challenging to translate to natural, genetically diverse animal populations, which limits their inference.

An example of natural fattening can be found in obligate seasonal hibernators such as ground squirrels, which gain fat mass during summer and use those fat reserves to hibernate for months at a time. However, studying the relationship between weight gain and the microbiome in wild animals comes with its own challenges due to numerous external factors that can influence microbiomes, such as fluctuating environmental conditions and diets. To minimize potentially confounding factors, our study worked with captive-born, wild-bred animals that show a strong annual cycle of fattening and hibernation that is maintained in captivity: thirteen-lined ground squirrels (*Ictidomys tridecemlineatus*). Thirteen-lined ground squirrels undergo annual winter hibernation, which is characterized by seven months of torpor bouts interspersed with regular, short periods of arousal (interbout arousal: IBA [7]).Torpor bouts are characterized by reduced metabolism and low body temperature [8], and squirrels neither eat nor drink during hibernation, resulting in substantial weight loss. Thus, to successfully hibernate, squirrels need to store fat rapidly during the 4–5 month active season prior to going into hibernation. Rapid fat storage in squirrels is a result of excessive eating, a behavior called hyperphagia early in the active season, and metabolic depression later in the active season [9].

The gut microbiome of 13-lined ground squirrels is dynamic through the annual cycle of feeding and fasting [7]. Along the length of the GI tract there are two general gut regions, the mucosa and the lumen. The lumen consists of the internal, transient part of the gastrointestinal tract and was found to be strongly influenced by diet in mice [10, 11]. The mucosal microbiome consists of microbes associated with the mucus lining that covers the intestinal epithelial layer [12]. The mucosal microbiome has less turnover than the lumen microbiome, and houses a unique microbial community that often includes mucus-degrading microorganisms [11]. The squirrel cecum mucosa houses a less diverse microbial community during hibernation than during the active season in spring and summer, but key microbial taxa remain constant [13]. Microbial diversity in the cecum lumen also increased during the active season compared to hibernation [7]. However, in contrast with the mucosal communities, large shifts in community composition were observed in the cecum lumen [7].

A study on another obligate hibernator species, the arctic ground squirrel (*Urocitellus parryii*), showed no change in microbiome composition over their short, intense active season, but did detect functional changes of the microbiome associated with diet [9]. Thirteen-lined ground squirrels have a longer active season than arctic ground squirrels, which presents a longer time frame for microbial change to occur. However, up to present, studies on 13-lined ground squirrels have focused on microbiome composition at single time points during the active season, prohibiting investigating the relationship between the microbiome and weight gain throughout the season. Since lethal sampling is required, multiple sampling time points would require several experimental groups of squirrels experiencing the same diet and environment during the entire sampling period. Here, we studied microbiome dynamics of 13-lined ground squirrels at four timepoints throughout the active season. In addition to studying the effect of time, we sequenced microbial communities two gut compartments: the cecum and the ileum. The cecum and ileum differ markedly in physiological structure that can affect microbial community dynamics. The cecum consists of a pouch where contents continually mix, while the ileum consists of a long tube with a unidirectional flow. Functionally, the cecum is involved in water absorption and absorption of nutrients freed up through bacterial fermentation, while the ileum’s main function is enzyme production and absorption of readily available nutrients. The physiological and functional differences between these gut compartments likely affect their microbial communities in both composition and abundance by creating different niches.

The aim of our study was to characterize microbiome dynamics in two gut compartments (cecum and ileum) of the gastrointestinal tract of 13-lined ground squirrels at pre-defined times during the pre-hibernation fattening period. We hypothesize that: (1a) microbial community composition overall shifts during the active season, due physiological changes associated with rapid fattening, (1b) Microbiome composition moves towards a Firmicutes-dominated community during the fattening period, as higher Firmicutes:Bacteroidetes ratios have been associated with weight gain in mammals, (2) gut microbiomes differ between the cecum and ileum due to differences in function and physiology, and (3) microbiome communities are more host-specific in the mucosal samples than the lumen samples as we expect lumen samples to be influenced more by transient microorganisms.

## Results

Squirrel body mass increased over the active season, and mass gain leveled off after week 14 (Additional file 1: Fig. S1; from [14]). In contrast to other studies [15], body mass at time of euthanasia of our squirrels did not significantly differ between sampling weeks (ANOVA: F_4,25_ = 0.116, p = 0.976), supporting the lack of weight gain observed during the latter part of our study. Despite the overall increase in body mass over most of the active season, caloric intake of the squirrels peaked early at week nine and decreased steadily after (Additional file 1: Fig. S1). Sequence numbers ranged from 2811–70,230 sequences/sample, with an average of 15,755 ± 13,101. Prior to rarefaction, we detected 1023 ASVs in our 89 samples, and 994 ASVs remained after rarefying to 2811 seqs/sample.

### Alpha and beta diversity

**Fig. 1:**
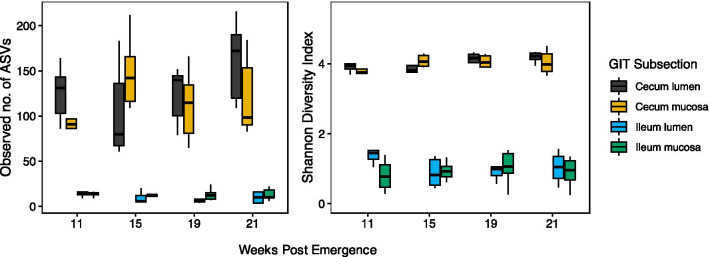
Observed number of ASVs and Shannon Alpha Diversity of four different subsections of the gastrointestinal tract of thirteen-lined ground squirrels sampled at 11–21 weeks after emerging from hibernation

Shannon’s H alpha diversity was significantly higher in the cecum compared to the ileum (Fig. 1; ANOVA: F_1,87_ = 669.5, p < 0.001), but not between mucosa and lumen samples (ANOVA: F_1,87_ = 0.016, p = 0.9), and number of weeks post-emergence (ANOVA: F_1,87_ = 0.42, p = 0.519). Observed number of ASVs followed the same pattern of significance.

**Fig. 2:**
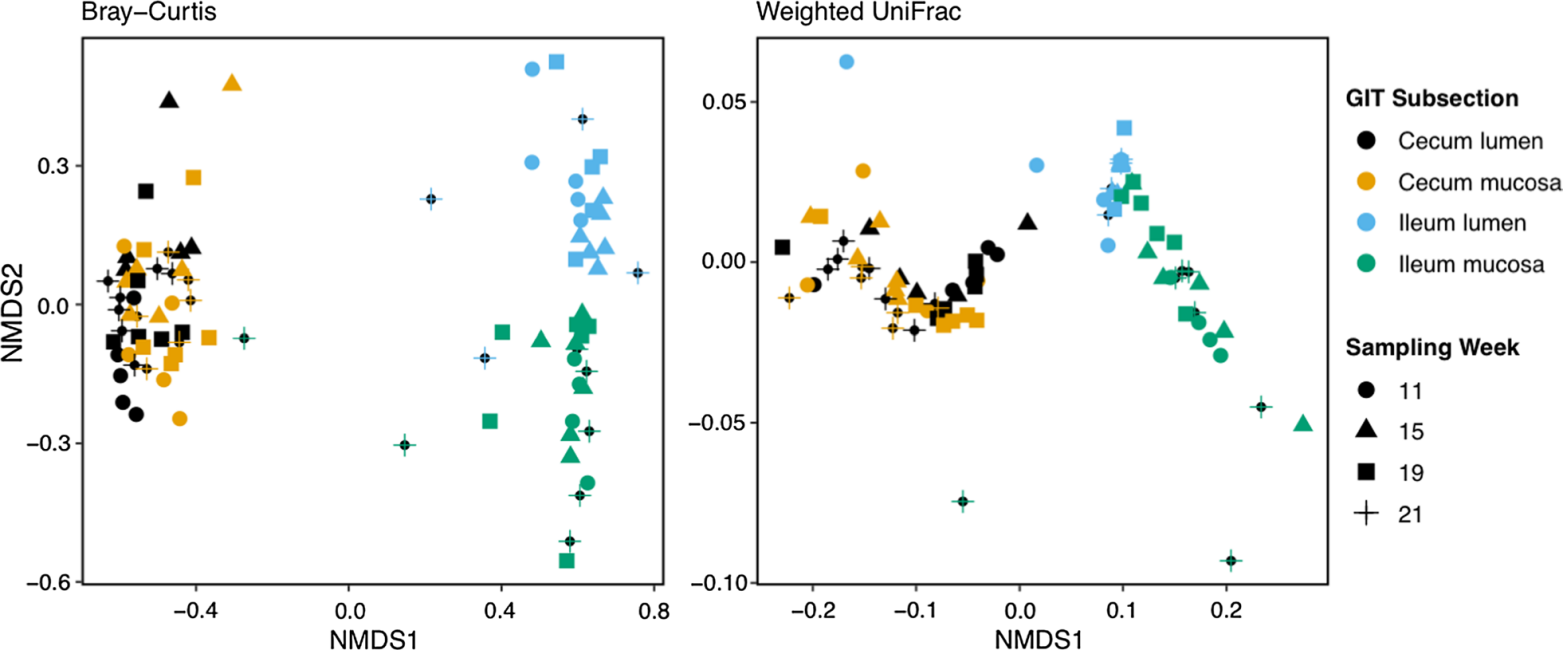
NMDS of Bray–Curtis and weighted UniFrac distance matrices of microbiomes of thirteen-lined ground squirrels. Colors represent different sections of the cecum and ileum, and shape represents number of weeks post emergence from hibernation

We reported results for the Bray–Curtis distance matrix in our manuscript and refer to Table 1 for Weighted UniFrac distance results. Unweighted UniFrac NMDS did not converge due to values exceeding the maximum stress levels. The NMDS showed a clear distinction between cecum and ileum samples, especially when using a Bray–Curtis distance matrix (Fig. 2). Distance to sample cloud centroid was significantly larger in the ileum samples compared to cecum samples (Betadispersion: F_1,87_ = 10.40, p < 0.001), which supports the visual assessment of the NMDS.

**Table 1:** PERMANOVA results for Bray–Curtis and Weighted UniFrac distance matrices for eight variables

	Bray–Curtis		Weighted UniFrac	
	R^2^	*p*	R^2^	*p*
Weeks post emergence	0.016	0.051	0.006	0.114
Compartment (cecum vs. ileum)	0.212	< 0.001	0.752	< 0.001
Tissue (mucosa vs. lumen)	0.047	< 0.001	0.008	0.053
Cecum only (mucosa vs. lumen)	0.001	0.995	0.032	0.189
Ileum only (mucosa vs. lumen)	0.271	< 0.001	0.248	< 0.001
Squirrel ID	0.027	0.002	0.006	0.110
Cecum only—squirrel ID	0.037	0.012	0.043	0.093
Ileum only—squirrel ID	0.028	0.314	0.004	0.908

Microbiome community composition was indeed significantly different between the cecum and ileum (PERMANOVA: R^2^ = 0.212, p < 0.001) and among sampling week (PERMANOVA: R^2^ = 0.016, p = 0.048). We detected significantly different microbial communities between the mucosa and lumen of the ileum (PERMANOVA: R^2^ = 0.271, p < 0.001), but not in the cecum (PERMANOVA: R^2^ = 0.01, p = 0.995). Interestingly, squirrel ID was a significant driver of microbiome composition in the cecum (PERMANOVA: R^2^ = 0.037, p = 0.012), but not in the ileum (PERMANOVA: R^2^ = 0.028, p = 0.314), suggesting that the cecum is more individually unique than the ileum.

### Community composition

**Fig. 3:**
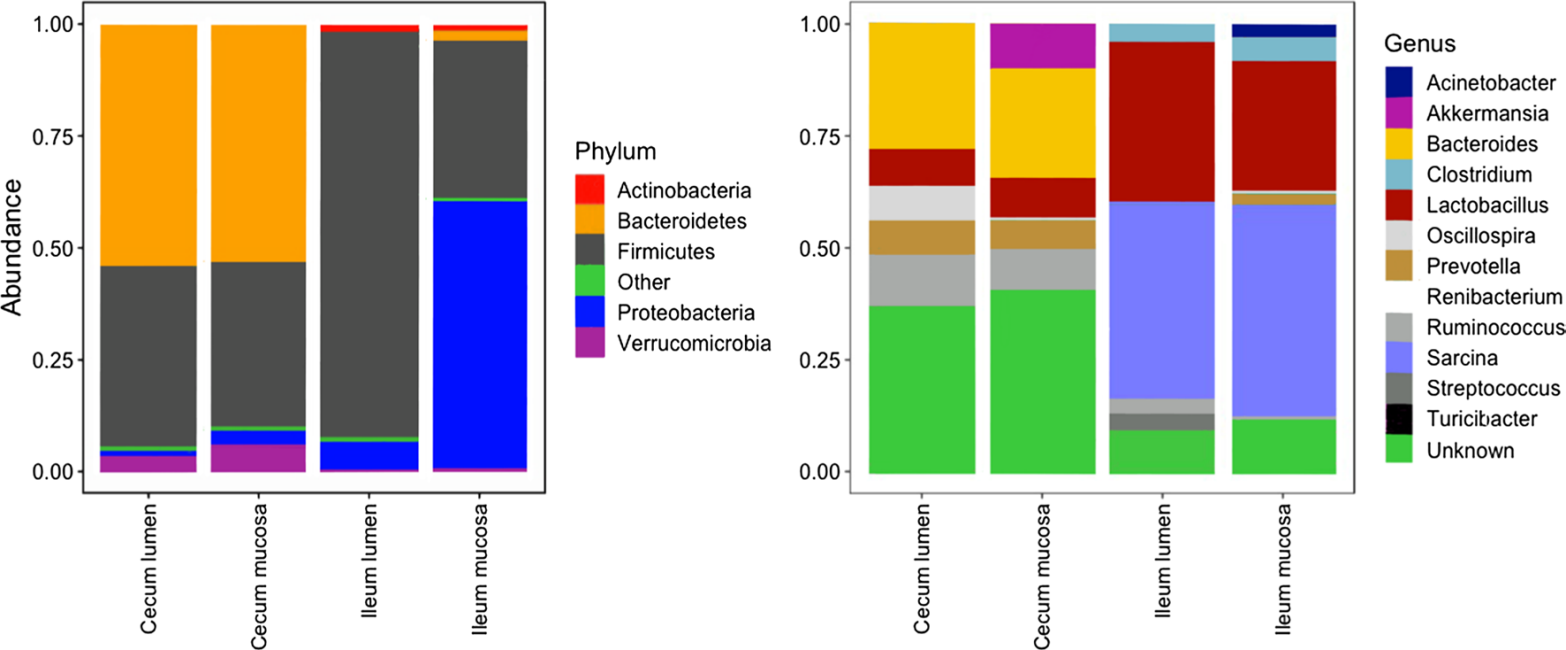
Relative sequence abundances of gut microbial communities in different subsections of the cecum and ileum of thirteen-lined ground squirrels at Phylum and Genus taxonomic levels. For clarity, we showed the top five most abundant Phyla, and the eight most abundant Genera per subsection

**Fig. 4:**
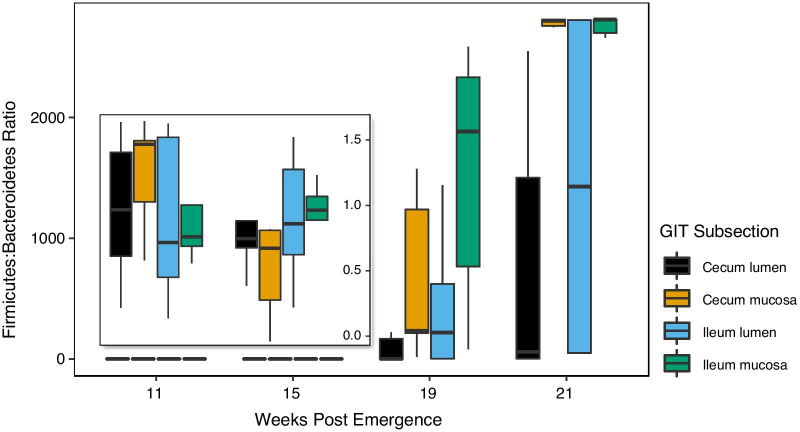
Ratio of sequences identified as Firmicutes to sequences identified as Bacteroidetes at four sampling weeks in four subsections of the gastrointestinal tract of thirteen-lined ground squirrels. Insert shows data for week 11 and 15 with an adjusted y-axis

We observed similar patterns in relative abundance between the cecum mucosa and lumen at phylum and genus level (Fig. 3, Additional file 2: Fig. S2). The genus *Akkermansia* was the second most abundant in both cecum subsections, but near absent in the ileum. The ileum lumen contained predominantly Firmicutes on a phylum level, but the mucosa was dominated by Proteobacteria. Within the Proteobacteria, the most abundant class and family were the Gammaproteobacteria and Enterobacteriaceae (Additional file 3: Fig. S3). On a genus level, *Sarcina* and *Lactobacillus* sp. dominated the ileum lumen and mucosa microbiomes. The lack of Proteobacteria genera in our abundance plots was due to the presence of a high number of different genera within the Proteobacteria all with relatively low numbers of sequences per genus. We detected an increase over time in the Firmicutes:Bacteroidetes ratio in all subsections, with the lowest increase in the cecum lumen (Fig. 4). The ileum mucosa had a higher ratio than the ileum lumen at all time points.

**Fig. 5:**
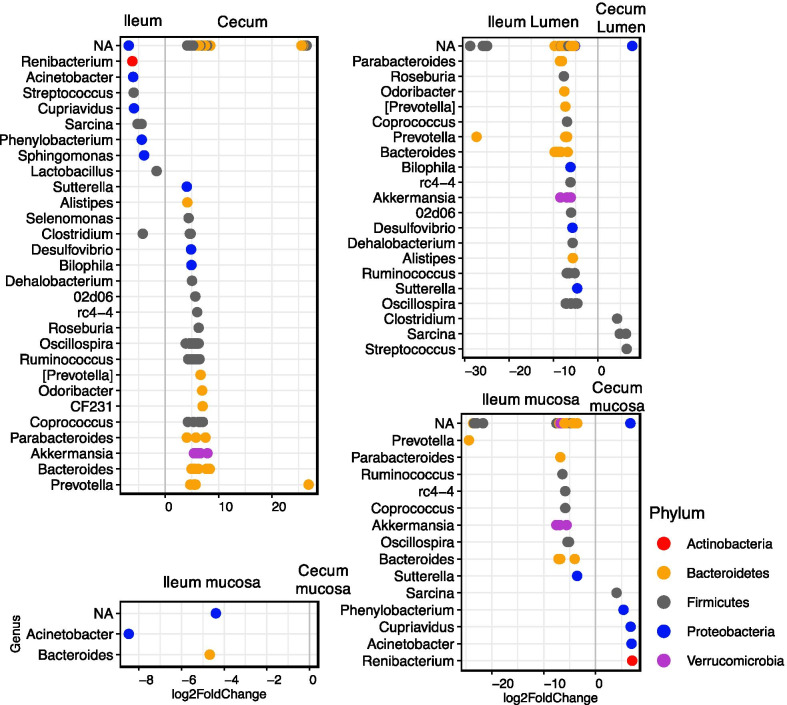
Differential bacterial genus abundance in different compartments and subsections of the gut of thirteen-lined ground squirrels. NA includes sequences that could not be confidently classified to genus level. Colors represent different phyla that the displayed genera belong in. Genera were considered differentially abundance if the adjusted p-value < 0.01

Consistent with our alpha diversity results, we detected more differentially abundant genera in the cecum compared to the ileum, most of which were part of the Bacteroidetes and Firmicutes Phyla (Fig. 5). We did not detect any differential abundant genera between the cecum lumen and mucosa. Only two identified genera were significantly different between the ileum mucosa and lumen, and all were more abundant in the mucosa: *Acinetobacter* and *Bacteroides*. *Akkermansia* sp. were significantly less abundant in samples from week 11 compared to week 15, 19, and 21 (Additional file 4: Fig. S4). We detected the opposite trend in *Prevotella* sp., which abundance decreased over time. Similarly, *Lactobacillus* sp. significantly decreased later in the active season.

**Fig. 6:**
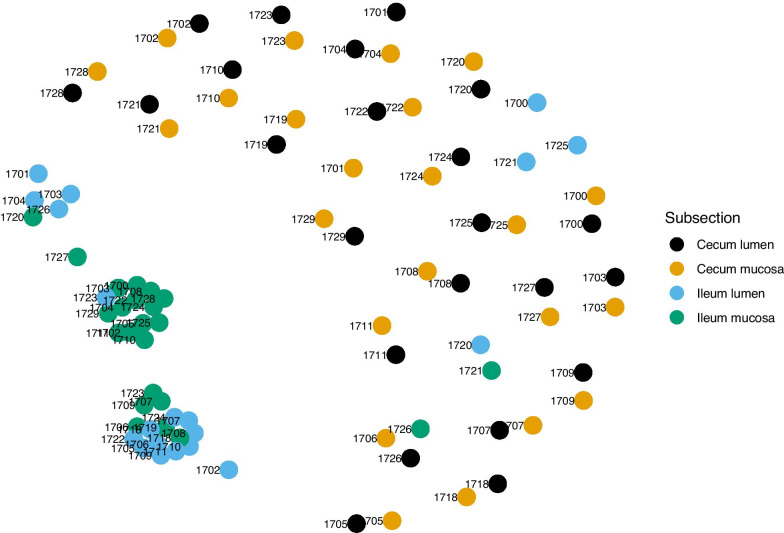
Similarity network of microbiomes samples collected from the cecum and ileum of thirteen-lined ground squirrels. Colors represent different sections of the cecum and ileum, and shape represents number of weeks post emergence from hibernation

### Network and phylogenetic analysis

In the cecum, mucosa and lumen samples grouped by individual squirrel (Fig. 6). Samples clustered by subsection in the ileum, and by individual squirrel in the cecum. Only one sample pair showed clustering by two different squirrel IDs (17–06 and 17–26). The associated mucosa and lumen samples from these two individuals did not cluster with any other samples and were thus not shown. Two cecum and three ileum samples did not cluster at a maximum ecological distance of 0.5.

**Fig. 7:**
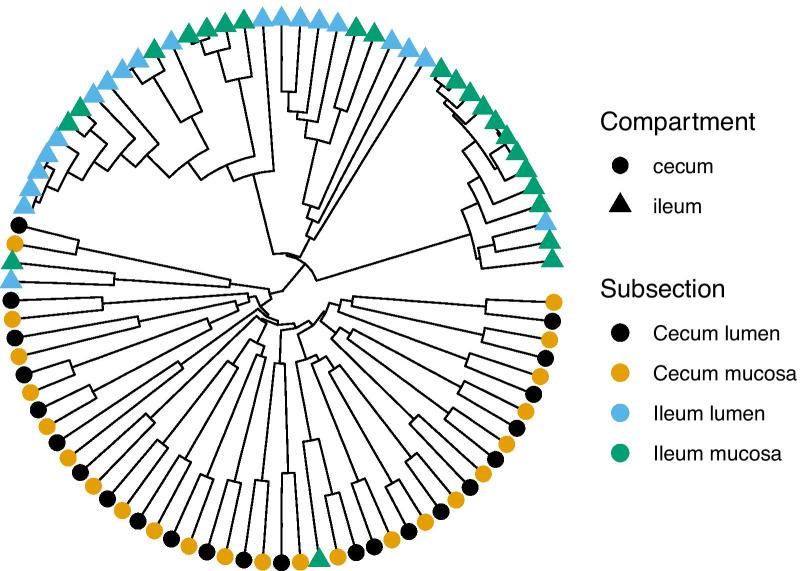
Phylogenetic tree of the Bray–Curtis distance matrix from microbial communities in the GIT of thirteen-lined ground squirrels. Tips represent individual samples and colors represent different subsection of the cecum and ileum

We detected two distinct clusters in our phylogenetic tree, effectively separating the ileum from the cecum samples (Fig. 7). Similar to the network (Fig. 6), cecum samples mostly clustered in mucosa and lumen pairs collected from the same individuals, while ileum samples did not cluster by individual squirrels. Two ileum mucosa and one ileum lumen samples clustered with the cecum samples for unknown reasons.

## Discussion

We detected significant differences in bacterial abundance and community composition between the cecum and ileum sections of the gastrointestinal tract of thirteen-lined ground squirrels over the duration of the active season. Contradictory to our hypothesis, we did not detect a difference in microbiome of these sections at different time points in the season. A possible reason for this result is that the first squirrels were sampled at 11 weeks post-hibernation, which allowed for an 11-week period for the gut microbiome to adapt to the physiological and behavioral changes associated with the shift from hibernation to active fattening. Carey et al. (2013) showed a significantly richer and more complex gut microbiome in thirteen-lined ground squirrels only two weeks post-hibernation compared to just prior to emergence. Similarly, gut microbiomes of arctic ground squirrels sampled three days post-hibernation already differed from hibernation microbiomes [16]. The gut microbiome of mammals is heavily influenced by diet [17–19], and can change rapidly in response to dietary changes such as the shift from not eating to eating post-emergence [10, 20]. It is likely that the squirrel gut microbiome had already reached a stable active season community by the time we first sampled.

The lumen and mucosal microbiome of thirteen-lined ground squirrels significantly differed in the ileum, but not the cecum. The cecum lumen and mucosa contained similar microbial communities, which could be attributed to the structure and function of this gut section. The cecum is a pouch between the ileum and colon that in small herbivorous mammals functions as a fermentative structure [21, 22], not unlike the bovine foregut. Bacteroidetes, and specifically *Bacteroides* (gen.), dominate the microbiome in both the cecum lumen and mucosa, and are known for their fermentation of plant-polysaccharides [23, 24]. Due to its sac-like structure, the cecal microbiome is likely less affected by rapid throughput of transient, diet-associated bacteria. The close and prolonged contact between the lumen and mucosa in the cecum could also lead to more mixing of the microbial communities and result in similar compositions, but data on retention time is absent at present.

We detected a significant effect of individual squirrel on the cecum microbiome. The cecum lumen and mucosa clustered by individual rather than by subsection, which was the opposite signature detected in the ileum. Ileum lumen and mucosa microbiomes were markedly different, which could be due to the larger influence of transient microbes. Functionally, the ileum is predominantly involved in absorption of vitamins, fatty acids, and bile acids [25]. We detected lower bacterial diversity in both the ileum lumen and mucosa than in the cecum, which matches results across different vertebrate taxa, such as birds [26], pigs [27, 28], and rodents [29, 30].

The lack of change in microbial communities in our study from week 11 to week 21 post-emergence was surprising as weight gain is generally associated with shifts in microbiome in mammals [31, 32]. However, the bulk of weight and fat mass gain had already occurred by week 11 post-emergence with only minor (but significant) increases after that point. A similar absence of a relationship between time and weight gain on the gut microbiome was also observed in arctic ground squirrels [9], indicating a potential insensitivity to change of the obligate hibernator microbiome. We did detect a possible increase in Firmicutes:Bacteroidetes ratios in mucosal communities of the cecum and ileum, but this increase was not large enough to be reflected in the overall community composition. A higher Firmicutes: Bacteroidetes ratio and a lower diversity have been associated with weight gain in humans and mice [1], although the absence of a correlation has also been observed (Reviewed in [33]).

## Conclusion

Overall, we identified specific bacterial communities associated with four gut sections of thirteen-lined ground squirrels. The large differences we found in microbiome communities of the squirrel cecum and ileum illustrated the effect micro-habitats and GI tract physiology can have on the gut microbiome. Additionally, we found little effect of sampling week on the gut microbiome and want to stress the future importance of frequent sampling early in the active season to capture the full active season dynamics. One caveat of our study was the use of female animals only, which limits our inference for the entire species. Female and males differ in physiology, especially pertaining to hormones, which could lead to different results than we found for female squirrels. In future studies, both sexes will be included to address this limitation. In addition, a next step for research focused on microbiome variation in hibernators would be to use additional meta-omics sequencing techniques to identify functional potential and changes throughout the squirrel annual cycle.

## Methods

### Study system and sampling

Twenty-four age-matched yearling female 13-lined ground squirrels were obtained from the University of Wisconsin Oshkosh squirrel colony after successful hibernation in captivity. Squirrels were housed in cages (10″ wide × 19″ long × 8″ tall) at ~ 20–22 °C with unlimited access to water. Light–dark cycles were adjusted regularly to match conditions in Oshkosh, WI (44° 01′ 27″ N, 88° 33′ 40″ W). Squirrels were fed Teklad Global 18% Protein Diet (#2018, Envigo, Madison, WI) ad libitum and received 1 tbsp (~ 6.5 g) of sunflower seeds once a week. Body mass for each squirrel was recorded weekly.

After being anesthetized with 4–5% isoflurane, squirrels were euthanized by decapitation at 11, 15, 19, and 21 weeks post-emergence from hibernation (n = 6 per timepoint). The GI tract was removed in its entirety. The cecum and ileum were cut open with sterile instruments and the luminal contents removed to a sterile tube. The tissue was rinsed with sterile saline and sterile slides were used to gently separate the mucosal layer from the underlying tissue. Samples were flash frozen in liquid nitrogen and stored at − 80 °C until DNA extraction.

### DNA extraction and sequencing

We extracted DNA from cecal and small intestine contents (mucosa and lumen; ~ 0.15 g/extraction) using Qiagen RNeasy PowerMicrobiome kits (Qiagen, Hilden, Germany) according to manufacturer protocols with omission of the DNase digestion step. DNA concentration and purity were measured with a Qubit flourometer (Invitrogen; Carlsbad, California, USA) and extractions were stored at − 80 °C. The V4 region of the 16S rRNA gene was PCR-amplified in triplicate using universal bacterial primers 515F (5′-GTGCCAGCMGCCGCGGTAA-3′) and 806R (5′-CAAGCAGAAGACGGCATACGAGAT-3′) with attached 8-bp Golay barcodes following to the Earth Microbiome Project protocol (available in the public domain at www.earthmicrobiome.org; [34]. Amplification success and purity was checked by gel electrophoresis. We pooled PCR products and cleaned DNA using the AxyPrep Mag PCR clean-up kit (Axygen; Union City, California, USA). Final DNA concentrations were determined using the Kapa Library Quantification Kit (Roche Sequencing Solutions Inc., Pleasanton, CA) and samples were combined in equimolar amounts. Libraries were sequenced paired-end (300 × 2 bp) using Illumina MiSeq platform (MiSeq v2 Reagent Kit).

### Sequence processing

We used the Quantitative Insights into Microbial Ecology 2 (QIIME2; v.2019.1) software for quality control and sequence analysis [35]. Sequences were demultiplexed and quality filtered using QIIME 2 (q2)-demux emp-paired plugin. Sequences were quality filtered and denoised using Deblur to identify all observed amplicon sequence variants (ASVs; [36]. ASVs that occurred less than 2 times were removed. We aligned ASVs using MAFFT [37], and constructed a phylogenetic tree with FastTree2 [38]. Taxonomy was assigned to ASVs in QIIME2 using the Naive Bayes classifier and the q2-feature-classify-sklearn plugin [39] against the Greengenes 13_8 99% OTUs reference database [40]. QIIME2 output was converted to a phyloseq object using the qza_to_phyloseq function from the *qiime2R* package [41]. We subsequently removed all non-target sequences (mitochondrial and chloroplast) from our dataset.

### Microbiome analysis and statistics

Samples were rarified to 2811 sequences/sample, which was the lowest number of sequences detected in a sample (Additional file 5: Fig. S5). We compared microbiome diversity for three variables: Compartment (cecum vs. ileum), Subsection (cecum lumen, cecum mucosa, ileum lumen, ileum mucosa), and Week (11, 15, 19, 21 weeks post emergence from hibernation). To investigate potential changes in body mass over the duration of our study, we compared weights of squirrels that were euthanized at different points of this study. Analysis of Variance (ANOVA) was used to assess statistical differences between sampling weeks.

All statistical analyses were conducted using R v. 4.0.2 [42]. To compare alpha diversity among sample variables, we calculated two diversity metrics: Observed number of ASVs (Observed) and Shannon’s H (Shannon). ANOVA was used to assess the differences in diversity in our variables. We calculated pairwise differences within variables using TukeyHSD tests with a Bonferroni correction for multiple comparisons.

We analyzed microbiome characteristics using the phyloseq package (v. 1.32.0; [43] and visualized results using the ggplot2 package [44]. To visualize betadiversity, we applied non-metric multidimensional scaling (NMDS) analysis to three distance matrices: Bray–Curtis [45], unweighted UniFrac, and weighted UniFrac [46]. To determine which variables (Compartment, Subsection, and Week) contributed to the most variation in microbiome composition, permutational multivariate analysis of variance (perMANOVA) was used with the *adonis2* function from the vegan package [47].

Bacterial abundances were summarized at the phylum and genus levels and bar plots of relative abundance were generated for all phyla and genera within Proteobacteria. Genera that represented less than one percent of the total relative abundance were excluded for clarity from taxonomic bar plots and charts. We used the *DESeq2* package (v. 1.28.1) in R to calculate the differentially abundant genera between different Compartments and Subsections of the squirrel GI tract [48]. *P*-values were corrected with the Benjamini and Hochberg false discovery rate for multiple testing [49]. Genera were identified as differentially abundant if the corrected *p* values < 0.01.

We created a sample-wise microbiome network from the Bray–Curtis distance matrix and a maximum ecological distance of 0.5 using the *phyloseq* and *igraph* package [50]. The maximum ecological distance refers to the largest difference allowed between two samples within the distance matrix to still be connected by an edge. For clarity we excluded all unconnected samples from our figure. Last, we constructed a circular phylogenetic tree of the Bray–Curtis distance matrix using the *ggtree* package in R [51], with tips representing individual samples. Tips and unique edges were colored by GIT subsection.

## Supplementary Information

**Additional file 1: Figure S1.** Body mass and caloric intake of thirteen-lined ground squirrels that were weighed weekly during the active season. Error bars represent standard error. Figure from Sonsalla et al. (2021).

**Additional file 2: Figure S2**. Relative abundance of classes, families, and genera within the Proteobacteria phylum divided over subsections of the Cecum and Ileum of thirteen-lined ground squirrels.

**Additional file 3: Figure S3**. Differential bacterial genus abundance in different weeks of sampling the cecum and ileum of thirteen-lined ground squirrels. NA includes sequences that could not be confidently classified to genus level. Colors represent different phyla that the displayed genera belong in. Genera were considered differentially abundance if the adjusted p-value < 0.01.

**Additional file 4: Figure S4.** Differential bacterial genus abundance in different weeks of sampling the cecum and ileum of thirteen-lined ground squirrels. NA includes sequences that could not be confidently classified to genus level. Colors represent different phyla that the displayed genera belong in. Genera were considered differentially abundance if the adjusted p-value < 0.01.

**Additional file 5: Figure S5.** Rarefaction curve of microbiome samples collected from thirteen-lined ground squirrels during the active season.

## Data Availability

Sequences were deposited in the NCBI database under Bioproject PRJNA676170 (Accession no. SAMN16757373- SAMN16757465).
