# Supplementary material for: Gut microbiome is affected by gut region but robust to host physiological changes in captive active-season ground squirrels
Source: Anim Microbiome. 2021 Aug 13;3:56. doi: 10.1186/s42523-021-00117-0 (PMC8361659; doi:10.1186/s42523-021-00117-0)
Supplement: Supplementary file 1 — Additional file 1: Figure S1. Body mass and caloric intake of thirteen-lined ground squirrels that were weighed weekly during the active season. Error bars represent standard error. Figure from Sonsalla et al. (2021). [file 42523_2021_117_MOESM1_ESM.docx]

Figure S1A) Body mass and caloric intake of thirteen-lined ground squirrels that were weighed weekly during the active season. Error bars represent standard error. Our study used the same individual squirrels that were studied by Sonsalla *et al.* (2021). B) Squirrel body mass at the time of euthanasia.
