# Supplementary material for: Gut microbiome is affected by gut region but robust to host physiological changes in captive active-season ground squirrels
Source: Anim Microbiome. 2021 Aug 13;3:56. doi: 10.1186/s42523-021-00117-0 (PMC8361659; doi:10.1186/s42523-021-00117-0)
Supplement: Supplementary file 2 — Additional file 2: Figure S2. Relative abundance of classes, families, and genera within the Proteobacteria phylum divided over subsections of the Cecum and Ileum of thirteen-lined ground squirrels. [file 42523_2021_117_MOESM2_ESM.docx]

S2. Relative phylum abundance of individual squirrels ordered by subsection and week.
