# Supplementary material for: Gut microbiome is affected by gut region but robust to host physiological changes in captive active-season ground squirrels
Source: Anim Microbiome. 2021 Aug 13;3:56. doi: 10.1186/s42523-021-00117-0 (PMC8361659; doi:10.1186/s42523-021-00117-0)
Supplement: Supplementary file 3 — Additional file 3: Figure S3. Differential bacterial genus abundance in different weeks of sampling the cecum and ileum of thirteen-lined ground squirrels. NA includes sequences that could not be confidently classified to genus level. Colors represent different phyla that the displayed genera belong in. Genera were considered differentially abundance if the adjusted p-value < 0.01. [file 42523_2021_117_MOESM3_ESM.docx]

Figure S3. Relative abundance of classes, families, and genera within the Proteobacteria phylum divided over subsections of the Cecum and Ileum of thirteen-lined ground squirrels.
