# Supplementary material for: Gut microbiome is affected by gut region but robust to host physiological changes in captive active-season ground squirrels
Source: Anim Microbiome. 2021 Aug 13;3:56. doi: 10.1186/s42523-021-00117-0 (PMC8361659; doi:10.1186/s42523-021-00117-0)
Supplement: Supplementary file 5 — Additional file 5: Figure S5. Rarefaction curve of microbiome samples collected from thirteen-lined ground squirrels during the active season. [file 42523_2021_117_MOESM5_ESM.docx]

Figure S5. Rarefaction curve of microbiome samples collected from thirteen-lined ground squirrels during the active season.
